# 5-Chloro-2-(4-fluoro­phen­yl)-7-methyl-3-methyl­sulfinyl-1-benzo­furan

**DOI:** 10.1107/S1600536813013172

**Published:** 2013-05-18

**Authors:** Hong Dae Choi, Pil Ja Seo, Uk Lee

**Affiliations:** a Department of Chemistry, Dongeui University, San 24 Kaya-dong, Busanjin-gu, Busan 614-714, Republic of Korea; b Department of Chemistry, Pukyong National University, 599-1 Daeyeon 3-dong, Nam-gu, Busan 608-737, Republic of Korea

## Abstract

In the title compound, C_16_H_12_ClFO_2_S, the 4-fluoro­phenyl ring makes a dihedral angle of 16.43 (4)° with the mean plane [r.m.s. deviation = 0.012 (1) Å] of the benzo­furan fragment. In the crystal, mol­ecules are linked by pairs of Cl⋯O contacts [3.1839 (12) Å] into inversion dimers, which are further packed into stacks along the *b* axis by weak C—H⋯O hydrogen bonds.

## Related literature

For background information and the crystal structures of related compounds, see: Choi *et al.* (2010*a* ▶,*b* ▶). For a review of halogen bonding, see: Politzer *et al.* (2007 ▶).
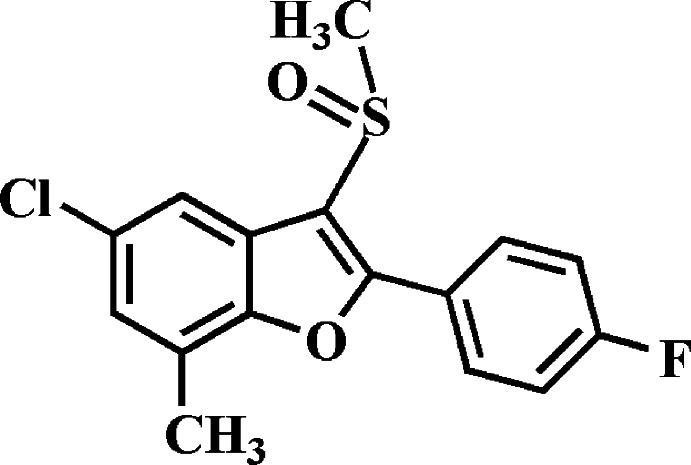


## Experimental

### 

#### Crystal data

C_16_H_12_ClFO_2_S
*M* *_r_* = 322.77
Triclinic, 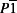

*a* = 7.5374 (3) Å
*b* = 9.7388 (3) Å
*c* = 10.7979 (4) Å
α = 106.902 (2)°
β = 90.605 (2)°
γ = 110.598 (2)°
*V* = 704.24 (4) Å^3^
*Z* = 2
Mo *K*α radiation
μ = 0.43 mm^−1^
*T* = 173 K
0.34 × 0.29 × 0.17 mm

#### Data collection

Bruker SMART APEXII CCD diffractometer
Absorption correction: multi-scan (*SADABS*; Bruker, 2009 ▶) *T*_min_ = 0.669, *T*_max_ = 0.746
13230 measured reflections
3505 independent reflections
3025 reflections with *I* > 2σ(*I*)
*R*_int_ = 0.029

#### Refinement

*R*[*F* ^2^ > 2σ(*F* ^2^)] = 0.033
*wR*(*F* ^2^) = 0.093
*S* = 1.04
3505 reflections
192 parameters
H-atom parameters constrained
Δρ_max_ = 0.38 e Å^−3^
Δρ_min_ = −0.30 e Å^−3^

### 

Data collection: *APEX2* (Bruker, 2009 ▶); cell refinement: *SAINT* (Bruker, 2009 ▶); data reduction: *SAINT*; program(s) used to solve structure: *SHELXS97* (Sheldrick, 2008 ▶); program(s) used to refine structure: *SHELXL97* (Sheldrick, 2008 ▶); molecular graphics: *ORTEP-3 for Windows* (Farrugia, 2012 ▶) and *DIAMOND* (Brandenburg, 1998 ▶); software used to prepare material for publication: *SHELXL97*.

## Supplementary Material

Crystal structure: contains datablock(s) global, I. DOI: 10.1107/S1600536813013172/xu5706sup1.cif

Structure factors: contains datablock(s) I. DOI: 10.1107/S1600536813013172/xu5706Isup2.hkl

Supplementary material file. DOI: 10.1107/S1600536813013172/xu5706Isup3.cml

Additional supplementary materials:  crystallographic information; 3D view; checkCIF report

## Figures and Tables

**Table 1:** Hydrogen-bond geometry (Å, °)

*D*—H⋯*A*	*D*—H	H⋯*A*	*D*⋯*A*	*D*—H⋯*A*
C9—H9*A*⋯O2^i^	0.98	2.52	3.2305 (17)	129

Symmetry code: (i) 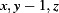.

**Fig. 1.**
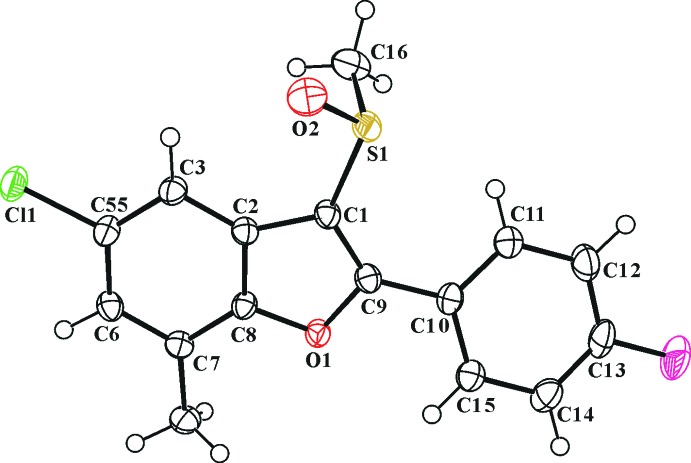
The molecular structure of the title compound with the atom numbering scheme. Displacement ellipsoids are drawn at the 50% probability level. H atoms are presented as small spheres of arbitrary radius.

**Fig. 2.**
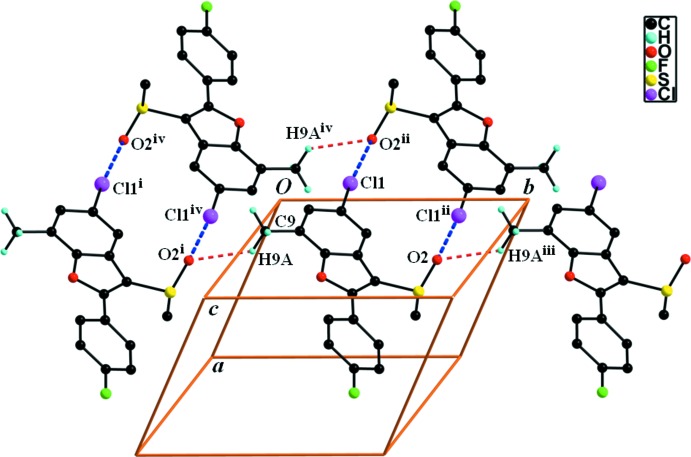
A view of the C—H···O and Cl···O interactions (dotted lines) in the crystal structure of the title compound. H atoms non-participating in hydrogen-bonding were omitted for clarity. [Symmetry codes: (i) x, y - 1, z; (ii) - x, - y + 1, - z; (iii) x, y + 1, z; (iv) -x, - y, - z.]

## supplementary crystallographic information

### Comment

As a part of our continuing study of 5-chloro-7-methyl-1-benzofuran derivatives containing [2-(4-chlorophenyl)-3-methylsulfinyl] (Choi *et al.*, 2010*a*) and [3-ethylsulfinyl-2-(4-fluorophenyl)] (Choi *et al.*, 2010*b*) substituents, we report herein the crystal structure of the title compound.

In the title molecule (Fig. 1), the benzofuran unit is essentially planar, with a mean deviation of 0.012 (1) Å from the least-squares plane defined by the nine constituent atoms. The dihedral angle formed by the 4-fluorophenyl ring and the mean plane of the benzofuran fragment is 16.43 (4)°. In the crystal structure (Fig. 2), molecules are connected by pairs of Cl···O halogen-bondings between the chlorine atom and the O atom of the S═O unit [Cl1···O2^ii^ = 3.1839 (12) Å, C4—Cl1···O2^ii^ = 173.77 (6)°] (Politzer *et al.*, 2007) into centrosymmetric dimers, which are further packed into stacks along the *b* axis by C—H···O hydrogen bonds (Table 1).

### Experimental

3-Chloroperoxybenzoic acid (77%, 291 mg, 1.3 mmol) was added in small portions to a stirred solution of 5-chloro-2-(4-fluorophenyl)-7-methyl-3-methylsulfanyl-1-benzofuran (368 mg, 1.2 mmol) in dichloromethane (30 mL) at 273 K. After being stirred at room temperature for 5 h, the mixture was washed with saturated sodium bicarbonate solution and the organic layer was separated, dried over magnesium sulfate, filtered and concentrated at reduced pressure. The residue was purified by column chromatography (hexane–ethyl acetate, 2:1 v/v) to afford the title compound as a colorless solid [yield 73%, m.p. 462–463 K; *R*_f_ = 0.51 (hexane–ethyl acetate, 2:1 v/v)]. Single crystals suitable for X-ray diffraction were prepared by slow evaporation of a solution of the title compound in acetone at room temperature.

### Refinement

All H atoms were positioned geometrically and refined using a riding model, with C—H = 0.95 Å for aryl and 0.98Å for methyl H atoms. *U*_iso_(H) = 1.2*U*_eq_(C) for aryl and 1.5*U*_eq_(C) for methyl H atoms. The positions of methyl hydrogens were optimized rotationally.

### Crystal data

**Table:**

C_16_H_12_ClFO_2_S	*Z* = 2
*M**_r_* = 322.77	*F*(000) = 332
Triclinic, *P*1	*D*_x_ = 1.522 Mg m^−^^3^
Hall symbol: -P 1	Melting point = 462–463 K
*a* = 7.5374 (3) Å	Mo *K*α radiation, λ = 0.71073 Å
*b* = 9.7388 (3) Å	Cell parameters from 5632 reflections
*c* = 10.7979 (4) Å	θ = 2.4–28.4°
α = 106.902 (2)°	µ = 0.43 mm^−^^1^
β = 90.605 (2)°	*T* = 173 K
γ = 110.598 (2)°	Block, colourless
*V* = 704.24 (4) Å^3^	0.34 × 0.29 × 0.17 mm

### Data collection

**Table:**

Bruker SMART APEXII CCD diffractometer	3505 independent reflections
Radiation source: rotating anode	3025 reflections with *I* > 2σ(*I*)
Graphite multilayer monochromator	*R*_int_ = 0.029
Detector resolution: 10.0 pixels mm^-1^	θ_max_ = 28.4°, θ_min_ = 2.0°
φ and ω scans	*h* = −10→10
Absorption correction: multi-scan (*SADABS*; Bruker, 2009)	*k* = −12→12
*T*_min_ = 0.669, *T*_max_ = 0.746	*l* = −14→14
13230 measured reflections	

### Refinement

**Table:**

Refinement on *F*^2^	Primary atom site location: structure-invariant direct methods
Least-squares matrix: full	Secondary atom site location: difference Fourier map
*R*[*F*^2^ > 2σ(*F*^2^)] = 0.033	Hydrogen site location: difference Fourier map
*wR*(*F*^2^) = 0.093	H-atom parameters constrained
*S* = 1.04	*w* = 1/[σ^2^(*F*_o_^2^) + (0.0509*P*)^2^ + 0.2009*P*] where *P* = (*F*_o_^2^ + 2*F*_c_^2^)/3
3505 reflections	(Δ/σ)_max_ = 0.001
192 parameters	Δρ_max_ = 0.38 e Å^−^^3^
0 restraints	Δρ_min_ = −0.30 e Å^−^^3^

### Special details

**Table:**

Geometry. All esds (except the esd in the dihedral angle between two l.s. planes) are estimated using the full covariance matrix. The cell esds are taken into account individually in the estimation of esds in distances, angles and torsion angles; correlations between esds in cell parameters are only used when they are defined by crystal symmetry. An approximate (isotropic) treatment of cell esds is used for estimating esds involving l.s. planes.
Refinement. Refinement of F^2^ against ALL reflections. The weighted R-factor wR and goodness of fit S are based on F^2^, conventional R-factors R are based on F, with F set to zero for negative F^2^. The threshold expression of F^2^ > 2sigma(F^2^) is used only for calculating R-factors(gt) etc. and is not relevant to the choice of reflections for refinement. R-factors based on F^2^ are statistically about twice as large as those based on F, and R- factors based on ALL data will be even larger.

### Fractional atomic coordinates and isotropic or equivalent isotropic displacement parameters (Å^2^)

**Table:**

	*x*	*y*	*z*	*U*_iso_*/*U*_eq_	
Cl1	−0.06877 (6)	0.24551 (4)	−0.05566 (3)	0.03248 (11)	
S1	0.31257 (5)	0.77726 (4)	0.43961 (3)	0.02701 (11)	
F1	0.57470 (16)	0.77811 (14)	1.05273 (9)	0.0546 (3)	
O1	0.18785 (14)	0.36631 (11)	0.48724 (9)	0.0232 (2)	
O2	0.16474 (17)	0.78460 (13)	0.35249 (12)	0.0385 (3)	
C1	0.25080 (19)	0.58204 (15)	0.43038 (13)	0.0224 (3)	
C2	0.15601 (19)	0.45115 (15)	0.31599 (13)	0.0218 (3)	
C3	0.0986 (2)	0.43012 (16)	0.18618 (13)	0.0243 (3)	
H3	0.1224	0.5150	0.1538	0.029*	
C4	0.0055 (2)	0.27967 (16)	0.10758 (13)	0.0247 (3)	
C5	−0.0335 (2)	0.15197 (16)	0.15232 (13)	0.0248 (3)	
H5	−0.1000	0.0510	0.0939	0.030*	
C6	0.02366 (19)	0.17061 (15)	0.28075 (13)	0.0230 (3)	
C7	0.11853 (19)	0.32240 (15)	0.35776 (13)	0.0213 (3)	
C8	0.26620 (19)	0.52538 (16)	0.53022 (13)	0.0229 (3)	
C9	−0.0088 (2)	0.03846 (16)	0.33323 (15)	0.0297 (3)	
H9A	0.1114	0.0228	0.3428	0.045*	
H9B	−0.1042	−0.0555	0.2728	0.045*	
H9C	−0.0550	0.0617	0.4185	0.045*	
C10	0.34642 (19)	0.59406 (16)	0.66751 (13)	0.0242 (3)	
C11	0.4761 (2)	0.74628 (18)	0.71675 (15)	0.0308 (3)	
H11	0.5137	0.8072	0.6601	0.037*	
C12	0.5504 (2)	0.8094 (2)	0.84709 (16)	0.0356 (3)	
H12	0.6359	0.9139	0.8813	0.043*	
C13	0.4979 (2)	0.7178 (2)	0.92584 (14)	0.0351 (4)	
C14	0.3715 (2)	0.5673 (2)	0.88172 (15)	0.0343 (3)	
H14	0.3382	0.5069	0.9390	0.041*	
C15	0.2938 (2)	0.50556 (18)	0.75203 (14)	0.0275 (3)	
H15	0.2040	0.4023	0.7201	0.033*	
C16	0.5173 (2)	0.79549 (19)	0.35447 (18)	0.0394 (4)	
H16A	0.4814	0.7153	0.2691	0.059*	
H16B	0.6164	0.7836	0.4052	0.059*	
H16C	0.5665	0.8976	0.3424	0.059*	

### Atomic displacement parameters (Å^2^)

**Table:**

	*U*^11^	*U*^22^	*U*^33^	*U*^12^	*U*^13^	*U*^23^
Cl1	0.0459 (2)	0.0299 (2)	0.01946 (17)	0.01465 (16)	−0.00394 (14)	0.00412 (14)
S1	0.0368 (2)	0.01850 (17)	0.02544 (19)	0.01193 (14)	0.00111 (14)	0.00443 (14)
F1	0.0573 (7)	0.0654 (8)	0.0226 (5)	0.0083 (6)	−0.0117 (5)	0.0057 (5)
O1	0.0287 (5)	0.0202 (5)	0.0203 (5)	0.0094 (4)	−0.0008 (4)	0.0055 (4)
O2	0.0474 (7)	0.0327 (6)	0.0430 (7)	0.0214 (5)	−0.0013 (5)	0.0151 (5)
C1	0.0264 (6)	0.0189 (6)	0.0216 (6)	0.0099 (5)	0.0005 (5)	0.0041 (5)
C2	0.0237 (6)	0.0194 (6)	0.0227 (6)	0.0094 (5)	0.0014 (5)	0.0056 (5)
C3	0.0309 (7)	0.0220 (7)	0.0220 (6)	0.0120 (6)	0.0015 (5)	0.0074 (5)
C4	0.0295 (7)	0.0265 (7)	0.0193 (6)	0.0131 (6)	0.0005 (5)	0.0056 (5)
C5	0.0268 (7)	0.0200 (6)	0.0245 (7)	0.0086 (5)	0.0001 (5)	0.0030 (5)
C6	0.0248 (6)	0.0204 (6)	0.0254 (7)	0.0104 (5)	0.0038 (5)	0.0071 (5)
C7	0.0241 (6)	0.0220 (6)	0.0193 (6)	0.0106 (5)	0.0008 (5)	0.0062 (5)
C8	0.0233 (6)	0.0211 (6)	0.0236 (6)	0.0092 (5)	0.0015 (5)	0.0046 (5)
C9	0.0391 (8)	0.0206 (7)	0.0304 (7)	0.0110 (6)	0.0050 (6)	0.0094 (6)
C10	0.0232 (6)	0.0284 (7)	0.0214 (6)	0.0125 (5)	0.0009 (5)	0.0051 (5)
C11	0.0301 (7)	0.0312 (8)	0.0261 (7)	0.0066 (6)	−0.0005 (6)	0.0078 (6)
C12	0.0315 (8)	0.0346 (8)	0.0293 (8)	0.0058 (6)	−0.0039 (6)	0.0018 (7)
C13	0.0325 (8)	0.0472 (10)	0.0194 (7)	0.0135 (7)	−0.0043 (6)	0.0035 (6)
C14	0.0362 (8)	0.0429 (9)	0.0261 (7)	0.0151 (7)	0.0017 (6)	0.0136 (7)
C15	0.0287 (7)	0.0293 (7)	0.0250 (7)	0.0120 (6)	0.0014 (5)	0.0078 (6)
C16	0.0399 (9)	0.0303 (8)	0.0474 (10)	0.0098 (7)	0.0114 (7)	0.0153 (7)

### Geometric parameters (Å, º)

**Table:**

Cl1—C4	1.7432 (14)	C6—C9	1.4975 (18)
Cl1—O2^i^	3.1839 (12)	C8—C10	1.4597 (19)
S1—O2	1.4848 (11)	C9—H9A	0.9800
S1—C1	1.7628 (13)	C9—H9B	0.9800
S1—C16	1.7863 (17)	C9—H9C	0.9800
F1—C13	1.3541 (17)	C10—C11	1.395 (2)
O1—C7	1.3749 (15)	C10—C15	1.399 (2)
O1—C8	1.3771 (16)	C11—C12	1.382 (2)
C1—C8	1.3669 (18)	C11—H11	0.9500
C1—C2	1.4443 (19)	C12—C13	1.370 (2)
C2—C7	1.3920 (18)	C12—H12	0.9500
C2—C3	1.3971 (18)	C13—C14	1.373 (2)
C3—C4	1.379 (2)	C14—C15	1.382 (2)
C3—H3	0.9500	C14—H14	0.9500
C4—C5	1.4004 (19)	C15—H15	0.9500
C5—C6	1.3884 (19)	C16—H16A	0.9800
C5—H5	0.9500	C16—H16B	0.9800
C6—C7	1.3845 (19)	C16—H16C	0.9800
			
C4—Cl1—O2^i^	173.77 (6)	C6—C9—H9B	109.5
O2—S1—C1	107.35 (7)	H9A—C9—H9B	109.5
O2—S1—C16	106.18 (8)	C6—C9—H9C	109.5
C1—S1—C16	97.46 (7)	H9A—C9—H9C	109.5
C7—O1—C8	106.81 (10)	H9B—C9—H9C	109.5
C8—C1—C2	107.29 (12)	C11—C10—C15	118.93 (13)
C8—C1—S1	127.30 (11)	C11—C10—C8	121.50 (13)
C2—C1—S1	125.16 (10)	C15—C10—C8	119.56 (13)
C7—C2—C3	119.26 (12)	C12—C11—C10	120.69 (14)
C7—C2—C1	104.92 (12)	C12—C11—H11	119.7
C3—C2—C1	135.81 (12)	C10—C11—H11	119.7
C4—C3—C2	116.44 (12)	C13—C12—C11	118.45 (15)
C4—C3—H3	121.8	C13—C12—H12	120.8
C2—C3—H3	121.8	C11—C12—H12	120.8
C3—C4—C5	123.30 (13)	F1—C13—C12	118.53 (15)
C3—C4—Cl1	118.71 (10)	F1—C13—C14	118.54 (15)
C5—C4—Cl1	117.99 (11)	C12—C13—C14	122.93 (14)
C6—C5—C4	121.04 (13)	C13—C14—C15	118.52 (14)
C6—C5—H5	119.5	C13—C14—H14	120.7
C4—C5—H5	119.5	C15—C14—H14	120.7
C7—C6—C5	114.78 (12)	C14—C15—C10	120.46 (14)
C7—C6—C9	121.71 (12)	C14—C15—H15	119.8
C5—C6—C9	123.49 (13)	C10—C15—H15	119.8
O1—C7—C6	124.18 (11)	S1—C16—H16A	109.5
O1—C7—C2	110.65 (12)	S1—C16—H16B	109.5
C6—C7—C2	125.16 (12)	H16A—C16—H16B	109.5
C1—C8—O1	110.30 (12)	S1—C16—H16C	109.5
C1—C8—C10	134.92 (13)	H16A—C16—H16C	109.5
O1—C8—C10	114.78 (11)	H16B—C16—H16C	109.5
C6—C9—H9A	109.5		
			
O2—S1—C1—C8	−140.84 (13)	C1—C2—C7—O1	−1.49 (15)
C16—S1—C1—C8	109.57 (14)	C3—C2—C7—C6	−1.2 (2)
O2—S1—C1—C2	32.66 (14)	C1—C2—C7—C6	178.13 (13)
C16—S1—C1—C2	−76.94 (13)	C2—C1—C8—O1	0.18 (15)
C8—C1—C2—C7	0.79 (15)	S1—C1—C8—O1	174.61 (10)
S1—C1—C2—C7	−173.79 (10)	C2—C1—C8—C10	179.48 (14)
C8—C1—C2—C3	179.96 (15)	S1—C1—C8—C10	−6.1 (2)
S1—C1—C2—C3	5.4 (2)	C7—O1—C8—C1	−1.10 (14)
C7—C2—C3—C4	0.47 (19)	C7—O1—C8—C10	179.45 (11)
C1—C2—C3—C4	−178.61 (15)	C1—C8—C10—C11	−17.7 (2)
C2—C3—C4—C5	0.6 (2)	O1—C8—C10—C11	161.60 (13)
C2—C3—C4—Cl1	−179.68 (10)	C1—C8—C10—C15	162.80 (15)
C3—C4—C5—C6	−1.1 (2)	O1—C8—C10—C15	−17.93 (18)
Cl1—C4—C5—C6	179.22 (10)	C15—C10—C11—C12	−0.5 (2)
C4—C5—C6—C7	0.36 (19)	C8—C10—C11—C12	179.93 (14)
C4—C5—C6—C9	−178.16 (13)	C10—C11—C12—C13	1.8 (2)
C8—O1—C7—C6	−178.00 (13)	C11—C12—C13—F1	178.17 (15)
C8—O1—C7—C2	1.63 (14)	C11—C12—C13—C14	−1.5 (3)
C5—C6—C7—O1	−179.67 (12)	F1—C13—C14—C15	−179.72 (14)
C9—C6—C7—O1	−1.1 (2)	C12—C13—C14—C15	0.0 (2)
C5—C6—C7—C2	0.8 (2)	C13—C14—C15—C10	1.3 (2)
C9—C6—C7—C2	179.31 (13)	C11—C10—C15—C14	−1.0 (2)
C3—C2—C7—O1	179.17 (11)	C8—C10—C15—C14	178.50 (13)

Symmetry code: (i) −*x*, −*y*+1, −*z*.

### Hydrogen-bond geometry (Å, º)

**Table:**

*D*—H···*A*	*D*—H	H···*A*	*D*···*A*	*D*—H···*A*
C9—H9*A*···O2^ii^	0.98	2.52	3.2305 (17)	129

Symmetry code: (ii) *x*, *y*−1, *z*.

## References

[bb1] Brandenburg, K. (1998). *DIAMOND* Crystal Impact GbR, Bonn, Germany.

[bb2] Bruker (2009). *APEX2*, *SADABS* and *SAINT* Bruker AXS Inc., Madison, Wisconsin, USA.

[bb3] Choi, H. D., Seo, P. J., Son, B. W. & Lee, U. (2010*a*). *Acta Cryst.* E**66**, o706. 10.1107/S1600536810006823 PMC2983503 21580444

[bb4] Choi, H. D., Seo, P. J., Son, B. W. & Lee, U. (2010*b*). *Acta Cryst.* E**66**, o886. 10.1107/S1600536810008627 PMC2983861 21580704

[bb5] Farrugia, L. J. (2012). *J. Appl. Cryst.* **45**, 849–854.

[bb6] Politzer, P., Lane, P., Concha, M. C., Ma, Y. & Murray, J. S. (2007). *J. Mol. Model.* **13**, 305–311. 10.1007/s00894-006-0154-7 17013631

[bb7] Sheldrick, G. M. (2008). *Acta Cryst.* A**64**, 112–122. 10.1107/S0108767307043930 18156677

